# Pancreatic cancer in type 1 and young-onset diabetes: systematic review and meta-analysis

**DOI:** 10.1038/sj.bjc.6603571

**Published:** 2007-01-16

**Authors:** R J Stevens, A W Roddam, V Beral

**Affiliations:** 1 Cancer Research UK Epidemiology Unit, University of Oxford, Richard Doll Building, Roosevelt Drive, Oxford OX3 7LF, UK

**Keywords:** pancreatic cancer, diabetes, young-onset diabetes

## Abstract

We conducted a systematic review of the risk of pancreatic cancer in people with type I and young-onset diabetes. In three cohort and six case–control studies, the relative risk for pancreatic cancer in people with (*vs* without) diabetes was 2.00 (95% confidence interval 1.37–3.01) based on 39 cases with diabetes.

An approximate two-fold increase in the risk of pancreatic cancer is found in people with diabetes (Everhart and Wright, 1995; Huxley *et al*, 2005). Most studies have either been restricted to people with type II diabetes, which account for 80–90% of cases, or have made no distinction between types of diabetes. The relationship between type I diabetes and pancreatic cancer, if any, is unknown. We conducted a systematic review of studies that assessed risk of pancreatic cancer in people with diabetes and recorded either whether the diabetes was type I or type II, or a surrogate for type I diabetes such as young age at onset.

## METHODS

### Data sources

Scopus (www.scopus.com) and Web of Knowledge (portal.isiknowledge.com) databases were searched for relevant articles. Briefly, we searched for articles that mentioned cancer or diabetes in the title, and both in the abstract or keywords, and that mentioned either pancreatic cancer or subtype of diabetes or both in the title, abstract or keywords. Scopus and Web of Knowledge identified 342 and 547 human studies, respectively, published up to 14 October 2006. Additional articles were identified by reference and citation searching from articles identified in the main search, and from meta-analyses of type II diabetes and pancreatic cancer (Everhart and Wright, 1995; Huxley *et al*, 2005).

### Study selection and data synthesis

Articles identified were assessed against the inclusion criteria: (i) sufficient information to estimate standardised incidence ratio (SIR) or odds ratio (OR); (ii) comparison of diabetic to nondiabetic subjects; (iii) subclassification of diabetes by type or age at onset. Studies restricted to patients with previous history of cancer or chronic pancreatitis were excluded.

For prospective studies, SIRs (none were found reporting hazard ratio) were aggregated by the general inverse variance method (Green and Higgins, 2006). For case–control studies, we extracted, or calculated where necessary (La Vecchia *et al*, 1994), cases and controls with and without type I or young-onset diabetes, and aggregated the resulting ORs by the Peto (‘O minus E’) method (Yusuf *et al*, 1985). In general, studies did not report age-adjusted ORs for type I diabetes. The combined summary result across cohort and case–control studies was calculated using the general inverse-variance method, regarding both SIRs and ORs as estimated relative risks (RRs).

## RESULTS

**Figure 1:**
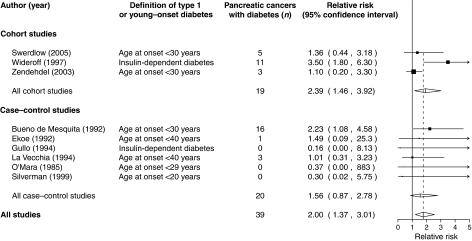
Relative risk of pancreatic cancer in type I or young-onset diabetes. Squares denote RR in individual studies and diamonds represent summary RRs (with sizes inversely proportional to CI width) and horizontal lines denote 95% CIs.

We identified three cohort and six case–control studies that reported pancreatic cancer by diabetes subtype (O'Mara *et al*, 1985; Bueno De Mesquita *et al*, 1992; Ekoe *et al*, 1992; Gullo *et al*, 1994; La Vecchia *et al*, 1994; Wideroff *et al*, 1997; Silverman *et al*, 1999; Zendehdel *et al*, 2003; Swerdlow *et al*, 2005). The overall RR for pancreatic cancer in young-onset or type I diabetes *vs* no diabetes was 2.00, with 95% confidence interval (CI) 1.37–3.01 (see Figure 1). Results for case–control and cohort studies were not dissimilar, based on the CIs.

## DISCUSSION

This study suggests an increased risk of pancreatic cancer in people with type I diabetes, based on 39 cases of pancreatic cancer in young-onset and type I diabetes, consistent with the 1.5- to two-fold increased risk that has been reported for type II diabetes (Everhart and Wright, 1995; Huxley *et al*, 2005).

Various hypotheses have been proposed for the elevated risk of pancreatic cancer in people with diabetes (Gapstur *et al*, 2000). However, studies of cancer, especially pancreatic cancer, have paid less attention to differences between type I and type II diabetes, but doing so may offer insights into possible mechanisms. In people with type II diabetes and people without diabetes, insulin resistance is compensated by increased insulin secretion by the *β*-cells, whereas in type I diabetes, insulin resistance may be present (DeFronzo *et al*, 1982) but *β*-cell function remains negligible. As our study indicates a similarly elevated risk in type I as in type II diabetes, this weighs against the involvement of *β*-cell activity in the aetiology of pancreatic cancer in diabetes.

The study is limited by the scarcity of published data, with only two studies reporting more than five exposed cases (with type I or young-onset diabetes). We did not have sufficient data to test heterogeneity statistically or otherwise examine sources of heterogeneity such as cohort demographics, methodology, and classification of diabetes type. In general, studies did not distinguish exocrine from endocrine pancreatic cancer. Most studies used age at onset of diabetes as a surrogate for diabetes type, but a study in Sweden found that by age 30, the age limit used by most of these studies, approximately 25% of cases of diabetes are type II (Berger *et al*, 1999). Considering only the studies that did not rely on age at onset would still yield a significant increased risk (RR 2.27, 95% CI 1.48–3.47), but this would be based on just two studies with only 11 pancreatic cancer cases with insulin-dependent diabetes.

The increased rate of pancreatic cancer in people diagnosed with type II diabetes may be partly attributable to reverse causality: that is, insulin resistance and diabetes may be induced by undiagnosed cancer or precancerous conditions of the pancreas (Gullo *et al*, 1994). However, the risk of pancreatic cancer is 1.5- to two-fold higher in type II diabetes even when impaired glucose tolerance is detected more than 5 years before onset of cancer (Everhart and Wright, 1995) or more than 10 years before onset of cancer (Huxley *et al*, 2005), suggesting that reverse causality does not, by itself, explain the association. The present study of type I diabetes could not directly examine the issue of reverse causality, but given the extreme infrequency of pancreatic cancer in people under 25, it is likely that type I diabetes precedes pancreatic cancer rather than the other way round.

The limited data currently available suggest that risk of pancreatic cancer is similarly elevated in people with both type I and type II diabetes. Further information, ideally from studies with large type I diabetic groups and nondiabetic reference groups, is needed to confirm this.
